# The interplay between metabolic homeostasis and neurodegeneration: insights into the neurometabolic nature of amyotrophic lateral sclerosis

**DOI:** 10.1186/s13619-015-0019-6

**Published:** 2015-08-27

**Authors:** S. T. Ngo, F. J. Steyn

**Affiliations:** 1 Queensland Brain Institute, The University of Queensland, St Lucia, Brisbane 4072 Australia; 2 School of Biomedical Sciences, The University of Queensland, St Lucia, Brisbane 4072 Australia; 3 Department of Neurology, Royal Brisbane and Women’s Hospital, Herston, Brisbane 4006 Australia; 4 University of Queensland Centre for Clinical Research, The University of Queensland, Herston, Brisbane, 4029 Australia

**Keywords:** Amyotrophic lateral sclerosis, Metabolism, Neurometabolism, Hyperexcitability, Ion channels, K_ATP_ channels

## Abstract

Amyotrophic lateral sclerosis (ALS) is a fatal, neurodegenerative disease that is characterized by the selective degeneration of upper motor neurons and lower spinal motor neurons, resulting in the progressive paralysis of all voluntary muscles. Approximately 10 % of ALS cases are linked to known genetic mutations, with the remaining 90 % of cases being sporadic. While the primary pathology in ALS is the selective death of upper and lower motor neurons, numerous studies indicate that an imbalance in whole body and/or cellular metabolism influences the rate of progression of disease. This review summarizes current research surrounding the impact of impaired metabolic physiology in ALS. We extend ideas to consider prospects that lie ahead in terms of how metabolic alterations may impact the selective degeneration of neurons in ALS and how targeting of adenosine triphosphate-sensitive potassium (K_ATP_) channels may represent a promising approach for obtaining neuroprotection in ALS.

## Introduction

Amyotrophic lateral sclerosis (ALS) is the most common form of motor neuron disease, initially described in 1869 by Jean-Martin Charcot [1]. ALS is a fatal, neurodegenerative disease in which the primary hallmark is the selective degeneration of upper motor neurons and lower spinal motor neurons. The loss of these motor neurons results in progressive paralysis of all voluntary muscles [2]. The underlying cause for ALS remains unknown, although many hypotheses to explain the selective death of upper and lower motor neurons have been proposed. Causative theories include abnormal protein function and RNA processing [3–7], mitochondrial dysfunction [8], non-cell autonomous death [9, 10], hyperexcitability [11, 12], excitotoxicity [13], and metabolic dysfunction [14]. Despite these theories, it is unlikely that ALS is caused by or results from any single one of these processes.

Approximately 10 % of ALS is defined as being familial, and the remaining 90 % of cases are considered sporadic, [15, 16]. Mutations in a number of genes including *C9orf72* [4, 7], *SOD1* [17], *TARDBP* [5, 18], and *FUS* [19, 20] cause familial ALS and contribute to sporadic ALS (reviewed in [15]). Interestingly, in line with the multifactorial nature of ALS, a recent modelling study by Al-Chalabi and colleagues suggests that in ALS, an underlying genetic susceptibility occurs in combination with environmental factors, which culminates in up to six exposures with the final exposure triggering the onset of disease [21]. Potential environmental risk factors that have been proposed to contribute to ALS include elite athleticism [22–24], β-methylamino-L-alanine (BMAA) [25, 26], pesticides [27, 28], and lifestyle factors (including smoking [29, 30], diet [31–35], and body mass index [36–41]) amongst many others (reviewed in [42]).

Evidence of metabolic dysfunction in ALS was reported throughout the 1970s and 1980s [43, 44]. Since that time, investigation into the contribution of the dysregulation in metabolic homeostasis to the pathogenesis of ALS has increased significantly. Numerous studies now indicate that ALS patients have impairments in whole body physiology and energy homeostasis, with data suggesting that an imbalance in energy metabolism appears to negatively influence the rate of progression of disease [14, 36–38, 40, 41, 45–62]. Should this be the case, attempts to offset energy deficits (e.g. through careful nutritional management [63]) to improve prognosis must take the metabolic state and underlying cause of metabolic perturbations of the person living with ALS into consideration. This review will focus on current research investigating the impact of impaired metabolic physiology in ALS and will consider prospects that lie ahead in terms of how metabolic alterations may impact the selective death of neurons in ALS.

## Metabolic homeostasis: the complex nature of balancing energy intake with energy expenditure

Metabolic homoeostasis and body composition requires balancing energy intake with energy expenditure. Seemingly simple in theory, the practical underpinnings of maintaining metabolic balance extend well beyond nutrient intake and absorption and resting metabolism and physical activity. Many fundamental regulators of metabolic physiology reside within the endocrine and neuroendocrine systems of the body. For example, orexigenic and anorexigenic neurons in the hypothalamus secrete neuropeptides that stimulate and inhibit appetite, respectively (reviewed in [64]), hormones secreted from the stomach and adipose control appetite (reviewed in [65]), pituitary-derived (e.g. growth hormone) and pancreatic hormones (e.g. insulin) play vital roles in modulating insulin action, glucose metabolism, free fatty acid flux, and body composition (reviewed in [66]), and the interplay between the neuroendocrine and endocrine systems can greatly influence physiological responses during periods of both positive and negative energy balance (reviewed in [67]). Not surprisingly, perturbation to endocrine and neuroendocrine processes typically results in the development of metabolic complications as is seen in type 2 diabetes and metabolic syndrome.

## Dysregulation of metabolic homeostasis in ALS: causes and consequences

In ALS patients, growth hormone deficiency [68], glucose intolerance [61], insulin resistance [43], hyperlipidemia [69], hypometabolism [46, 70–72], hypermetabolism [47, 48, 54, 72], and reduced body mass index (BMI) throughout the course of disease [36, 37, 41, 57, 59] are telling signs of the existence and progressive worsening of dysregulated metabolic homeostasis. These observations have sparked attempts to identify the underlying cause and the consequences of metabolic perturbations in ALS.

### ALS-causing genes and metabolism

The underlying cause of defective metabolic homeostasis in ALS remains to be fully determined. Mutations in or altered expression of ALS-associated genes in mice, cell lines, and humans are often coupled with metabolic abnormalities. In mice expressing SOD1^G86R^ or SOD1^G93A^ mutations, hypermetabolism and defects in glucose metabolism are observed [14, 60]. Deletion of *TARDP* (TDP-43) in adult mice results in weight loss, depletion of fat mass, and rapid death [73]. By contrast, overexpression of TDP-43 in mice (TDP-43^A315T^) results in increased fat deposition and hypertrophy of adipocytes [74]. When overexpressed in mouse skeletal muscle, TDP-43 drives an increase in the steady state expression of Tbc1d1, a Rab-GTPase-activating protein. Increased Tbc1d1 expression is thought to reduce insulin-stimulated translocation of the Glut4 transporter from tubulovesicular structures adjacent to the Golgi complex and from vesicles throughout the cytoplasm to the cell surface, impairing insulin-mediated glucose uptake [74]. Moreover, overexpression of human TDP-43 in mice underpins morphological abnormalities during mitochondrial formation [75, 76]. When considering *FUS* mutations, mass spectrometry analysis of protein interactions in HEK293 cells overexpressing mutant *FUS* associated with juvenile ALS demonstrate greater interactions with mitochondrial enzymes and proteins involved in glucose metabolism [77]. Not surprisingly, exogenous expression of mutant *FUS* in HEK293 and SH-SY5Y cells leads to a significant reduction in cellular adenosine triphosphate (ATP) production [77]. Finally, humans with ALS who harbour the *C9orf72* repeat expansion exhibit hypometabolism in numerous brain regions when compared to sporadic ALS patients [70]. Collectively, results indicate that the expression of ALS-associated genes *SOD1*, *TARDP*, *FUS*, and *C9orf72* is tightly linked to processes that are involved in regulating lipid and glucose homeostasis, mitochondrial formation, and ATP production. The presentation of metabolic defects in parallel with ALS-causing gene mutations point to the possible existence of a genetic predisposition to metabolic abnormalities in ALS and suggest a potential integral role for metabolic factors in regulating the progression and development of ALS.

## Targets of dysregulated metabolic homeostasis in ALS: the endocrine organs

Pristine physiological responses that occur throughout the body in response to metabolic pressures serve to ensure optimal metabolic flux. In turn, this sustains favourable responses to the metabolic demands of disease, thereby enhancing the likelihood for survival. Interestingly, altered metabolic homeostasis in ALS presents at the whole body level, and some of the targets that are affected in ALS are major endocrine organs that play crucial roles in regulating glucose and free fatty acid flux. We will briefly consider the adipose tissue, liver and muscle as critical metabolic organs that modulate homeostatic responses during the progression of ALS.

### Adipose

Adipose triglycerides represent the largest energy reserve in the human body. Within all cell types, triacylglycerols are stored as cytoplasmic lipid droplets or fat droplets that are enclosed by a monolayer of phospholipids and hydrophobic proteins. Fatty acids that arise from the breakdown of triacylglycerols play crucial roles in membrane biosynthesis, signal transduction, and energy production. Importantly, fatty acids that are derived from adipocyte triacylglycerols and released into circulation are in general, the primary regulators of fatty acid metabolism. Thus, in normal physiology, the maintenance of metabolic homeostasis is critically dependent on the flux between the uptake and storage of lipids (lipogenesis) during periods of positive energy balance and the breakdown and release of lipids (lipolysis) from adipocytes during periods of negative energy balance (reviewed in [78]).

It was first noted in the 1970s that ALS patients have larger subcutaneous fat cells [44], and it has been suggested that defects in carbohydrate metabolism and increased serum triglycerides in ALS patients might be somewhat related to this enlargement of subcutaneous fat cells. More recently, increased expression of a number of fat-derived cytokines (adipokines) that are associated with metabolic disease has been observed in ALS patients [79]. While the significance of these changes remains to be defined, there is evidence to show that the regulation of lipolytic processes to maintain metabolic flux could be key to promoting a survival advantage in ALS. In 2008, Dupuis et al. presented evidence to show that increased low-density lipoprotein:high-density lipoprotein ratio was associated with extended survival in ALS [69]. Subsequent to this, elevated serum triglycerides [51], higher palmitoleate and blood cell palmitoleate:palmitate ratio [80], and higher BMI (commonly used as a measure of increased body “fatness”) have been linked to improved survival in ALS [36, 37, 41, 57–59]. Moreover, Lindauer and colleagues have demonstrated a favourable relationship between subcutaneous adiposity and survival in ALS patients [81]. Thus, current studies suggest that the availability and mobilization of lipids from larger subcutaneous adipose stores into circulation may play a fundamental role in modulating the course of disease. In this regard, a greater capacity to mobilize lipids may favourably impact disease progression. The mechanisms by which increased fat mass or increased movement of lipids into circulation exerts beneficial effects in ALS remain to be determined, but it is plausible that the availability of excess fatty acids may assist in the provision of an alternative metabolic substrate to meet energy demand in ALS.

### Liver

The liver is an essential endocrine organ that regulates lipogenesis, gluconeogenesis, and cholesterol metabolism; it is a major site at which carbohydrates, proteins, and lipids are synthesized, metabolized, stored, and redistributed. Under fed states, the liver stores glycogen and triglyceride (which is later redistributed to adipose). In the fasted state, the liver releases glucose (formed via gluconeogenesis) and ketone bodies (produced from fatty acids). Influenced by glucose, insulin, and glucagon, liver carbohydrate and fatty acid metabolism orient metabolic fluxes towards energy storage or substrate release (reviewed in [82]).

Ultrastructural abnormalities in the liver [83–86], fatty acid infiltration into the liver [86], and mild liver dysfunction have been observed in ALS patients [86]. More recently, hepatic steatosis has been reported to be a frequent occurrence in ALS [69, 87]. While the discussions surrounding the prognostic and metabolic implications of hepatic steatosis in ALS remain open, abnormal insulin-like growth factor-1 (IGF-1) axis function alongside lipid redistribution in SOD1^G93A^ mice [88], and dysregulation of lipid metabolism in response to genetic ablation of TDP-43 in mice [73] provide a foundation upon which the beneficial effects of altered hepatic lipid metabolism in ALS can be explored.

### Skeletal muscle

**Fig. 1:**
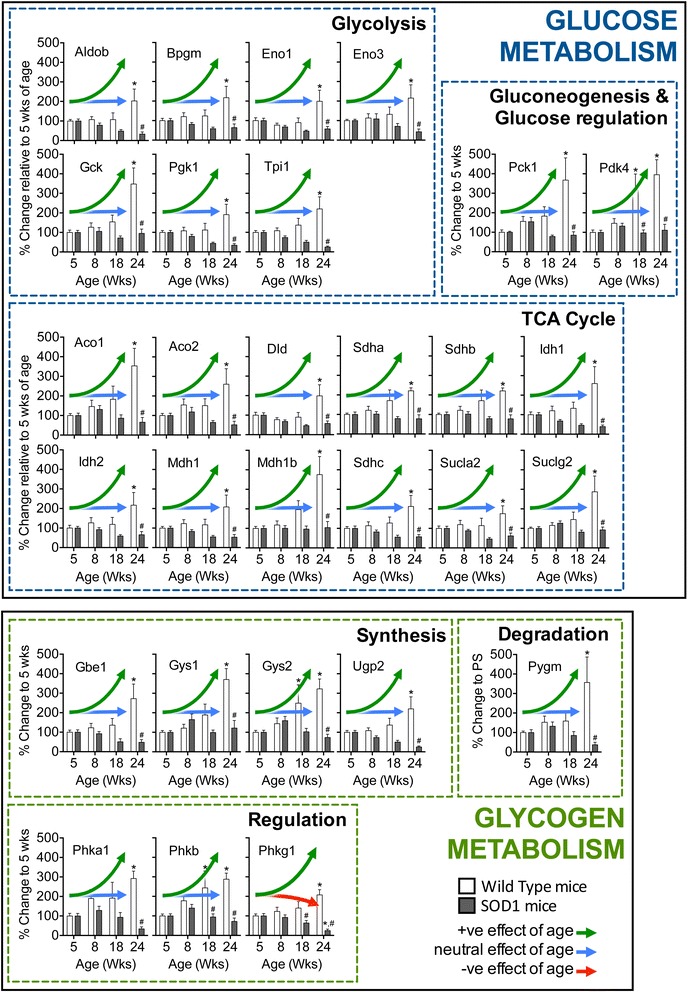
Expression of glucose and glycogen metabolism genes in the skeletal muscle of wild-type and SOD1^G93A^ mice. Compared to non-transgenic wild-type mice (*white bars*), the expression of glucose and glycogen metabolism genes in the skeletal muscle of SOD1^G93A^ mice (*black bars*) does not increase over the assessed period of muscle growth. Disease stages by age: pre-symptomatic (5 weeks), onset (8 weeks), mid-stage (18 weeks), and end-stage (24 weeks). *Green upward arrows* illustrate a significant effect (*p* < 0.05) of age following analysis by two-way ANOVA. *Blue arrows* represent no effect of age (*p* > 0.05) following analysis by two-way ANOVA. For SOD1^G93A^ mice, relative expression of *Phkg1* mRNA declined with age (illustrated by *red downward arrow*). The effect of age on gene expression was further interrogated using multiple comparison assessment with Bonferroni post hoc analysis; *significant differences (*p* < 0.05) at 8, 18, and 24 weeks of age when compared to 5 weeks of age. An effect of genotype within each age (5, 8, 18, and 24) was interrogated using multiple comparison assessment with Bonferroni post hoc analysis; ^#^significant (*p* < 0.05) differences between WT and SOD1^G93A^ mice at 5, 8, 18, or 24 weeks of age (*n* = 6 mice/group). Data presented as mean ± SEM. Gene descriptions, symbols, UniGene identifiers, and NCBI reference sequences (NCBI RefSeq) are provided in Table 1

Skeletal muscle is a major consumer of glucose and thus plays a fundamental role in the maintenance of glucose homeostasis and carbohydrate metabolism. Skeletal muscle is dependent upon small quantities of blood glucose during periods of rest or fasting. However, after insulin stimulation, the need for blood glucose in the skeletal muscle increases to approximately 75 % of that required by the body [89, 90]. Given its metabolically demanding nature, it has been proposed that metabolic defects in ALS originate from the skeletal muscle [91]. In support of this, muscle-restricted expression of the superoxide dismutase 1 (SOD1) gene causes muscle atrophy via oxidative damage and mitochondrial dysfunction [91, 92], and muscle restricted mitochondrial dysfunction drives motor neuron degeneration [93]. Moreover, in ALS skeletal muscle, structural and functional abnormalities in mitochondria [94–96], impaired glucose use and oxidative mitochondrial metabolism [60, 97–100], defective activity of respiratory complexes I and IV [95, 96, 101], and reduced cellular ATP [97] exist. More recently, using the SOD1^G86R^ mouse model of ALS, Palamuic and colleagues demonstrate that skeletal muscle mitochondrial dysfunction and denervation in ALS likely occurs due to a decreased ability to generate energy via glucose metabolism [60]. Consistent with this, our analysis of the skeletal muscle from the SOD1^G93A^ mouse model of ALS (B6.Cg-Tg(SOD1-G93A)1Gur/J; all SOD1^G93A^ mice had ≥ 25 copies of the SOD1 gene) using a mouse Glucose Metabolism RT2 Profiler PCR Array (PAMM-006Z, QIAGEN, Germany, strictly adhering to supplied protocols and guidelines) illustrate altered expression of a number of genes critically involved in the processes that regulate muscle glucose metabolism (listed in Table 1), starting at disease onset (8 weeks of age) and continuing through to mid-stage (18 weeks of age) and end-stage (24 weeks of age) of disease (Fig. 1). Compared to non-transgenic littermate control mice, we observed a marked decrease in genes central to all processes that are associated with glucose metabolism (including glycolysis, tricarboxylic acid (TCA) cycle, gluconeogenesis, and glucose regulation) and glycogen metabolism (including glycogen synthesis, regulation, and degradation). We observed a significant reduction in mRNA expression for the majority of target genes in the skeletal muscle of SOD1^G93A^ mice when compared to the progressive rise in gene expression that is normally observed in non-transgenic wild-type mice during the first 3 months of age (when muscle growth is occurring [102]). Observations suggest that metabolic processes that underpin the establishment of glucose use by muscle in ALS may be compromised, potentially reflecting the disease pathology. Whether altered expression patterns of glucose and glycogen metabolism genes are due to the overexpression of the human SOD1 gene, which itself is proposed to induce ALS-like pathologies observed in SOD1 mice [103–106], remains to be determined.

**Table 1:** Gene descriptions and identifiers for data described in Fig. 1

Symbol	Description	Gene symbol	UniGene identifier	NCBI RefSeq
Aco1	Aconitase 1	AI256519, Aco-1, Irebp, Irp1	Mm.331547	NM_007386
Aco2	Aconitase 2, mitochondrial	Aco-2, Aco3, D10Wsu183e	Mm.154581	NM_080633
Aldob	Aldolase B, fructose-bisphosphate	Aldo-2, Aldo2, BC016435, MGC36398	Mm.482116	NM_144903
Bpgm	2,3-Bisphosphoglycerate mutase	AI323730, AL022789, C86192	Mm.28263	NM_007563
Dld	Dihydrolipoamide dehydrogenase	AI315664, AI746344	Mm.3131	NM_007861
Eno1	Enolase 1, alpha non-neuron	0610008l15, AL022784, Eno-1, MBP-1, MGC103111, MGC107267	Mm.70666	NM_023119
Eno3	Enolase 3, beta muscle	Eno-3	Mm.251322	NM_007933
Gbe1	Glucan (1,4-alpha-), branching enzyme 1	2310045H19Rik, 2810426P10Rik, D16Ertd536e	Mm.396102	NM_028803
Gys1	Glycogen synthase 1, muscle	Gys3, MGS	Mm.275654	NM_030678
Gys2	Glycogen synthase 2	BC021322, LGS, MGC29379	Mm.275975	NM_145572
Idh1	Isocitrate dehydrogenase 1 (NADP+), soluble	AI31485, AI788952, E030024J03Rik, Id-1, Idh-1, Idpc, MGC115782	Mm.9925	NM_010497
Idh2	Isocitrate dehydrogenase 2 (NADP+), mitochondrial	E430004F23, IDPm, Idh-2	Mm.246432	NM_173011
Mdh1	Malate dehydrogenase 1, NAD (soluble)	B230377B03Rik, D17921, MDH-s, MDHA, Mor-2, Mor2	Mm.212703	NM_008618
Mdh1b	Malate dehydrogenase 1B, NAD (soluble)	1700124B08Rik, AV255588	Mm.30494	NM-029696
Pck1	Phosphoenolpyruvate carboxykinase 1, cytosolic	AI265463. PEPCK, Pck-1	Mm.266867	NM_011044
Pdk4	Pyruvate dehydrogenase kinase, isoenzyme 4	AV005916	Mm.235547	NM_013743
Pgk1	Phosphoglycerate kinase 1	MGC118097, Pgk-1	Mm.336205	NM_008823
Phka1	Phosphorylase kinase alpha 1	5330411D17, 9830108K24Rik, Phka	Mm.212889	NM_173021
Phkb	Phosphorylase kinase beta	AI462371, MGC62514	Mm.237296	NM_199446
Phkg1	Phosphorylase kinase gamma 1	Phkg	Mm.3159	NM_011079
Pygm	Muscle glycogen phosphorylase	AI115133, PG	Mm.27806	NM_011224
Sdha	Succinate dehydrogenase complex, subunit A, flavoprotein (Fp)	1500032O14Rik, 2310034D06Rik, 4921513A11, C81073, FP, SDH2, SDHF	Mm.158231	NM_023281
Sdhb	Succinate dehydrogenase complex, subunit B, iron sulfur (Ip)	0710008N11Rik	Mm.246965	NM_023374
Sdhc	Succinate dehydrogenase complex, subunit C, integral membrane protein	0610010E03Rik, AI316496, AU019277, MGC103103	Mm.198138	NM_025321
Sucla2	Succinate-coenzyme A ligase, ADP-forming, beta subunit	4930547K18Rik	Mm.38951	NM_011506
Suclg2	Succinate-coenzyme A ligase, GDP-forming, beta subunit	AF171077, AW556404, D6Wsu120e, MGC91183	Mm.371585	NM_011507
Tpi1	Triosephosphate isomerase 1	AI255506, Tpi, Tpi-1	Mm.4222	NM_009415
Ugp2	UDP-glucose pyrophosphorylase 2	MGC38262	Mm.28877	NM_139297

When glucose is not used for energy in the skeletal muscle, fatty acids [107] and ketones (which are a by-product of the metabolism of fat) can fuel ongoing energy demand [108]. Thus, it is not surprising that observations from SOD1^G86R^ mice identify increased peripheral clearance of lipids in response to supplementation with a high-fat diet [53]. Whether these measures of peripheral lipid clearance reflect an underlying physiological response to replace atrophic muscle with fat, fat accumulation due to denervation of muscle fibres, or fat/ketone transport into muscle for use as an alternative energy substrate remains unknown. Recent observations demonstrating an increase in the expression of genes that are critical in regulating fat metabolism in the skeletal muscle prior to denervation and improved endurance exercise performance in SOD1^G86R^ mice are congruent with the notion that there is a switch in energy substrate preference in the skeletal muscle from glucose towards fat [60]. While these data are convincing in proposing that reduced glucose metabolism in the skeletal muscle contributes to ALS pathophysiology (and reported fatigue [109]), muscle weakness in ALS is ultimately due to the loss of innervation from the dying neuron.

## Central hypermetabolism and hypometabolism: implications for neuronal death

A number of in vivo and in vitro studies have investigated brain or neuronal metabolism to provide insight into how the metabolic profile of neural cells might be associated with ALS neuropathology. Brain hypermetabolism has been observed in bilateral amygdalae, midbrain, pons, cerebellum, bilateral occipital cortex, globus pallidus, left inferior temporal cortex, temporal pole, and the hippocampus [45, 70, 72]. Given that this hypermetabolism has been attributed to the local activation of glial cells, it is likely that neurons in these brain regions are subjected to an environment that promotes non-cell autonomous death through the expression of mutant SOD1 [9, 10] or an α2-Na/K ATPase/α-adducin complex [110] in astrocytes. Brain hypometabolism (decreased use of glucose) is observed in frontal, motor, and occipital cortices, right insula, anterior and posterior cingulate, precuneus, inferior parietal lobe, caudate, thalamus, putamen, and the left frontal and superior temporal cortex of ALS patients [45, 70–72, 111, 112]. In addition, reduced glucose use has also been reported to occur in the spinal cords of SOD1^G93A^ mice [113]. Thus, it is plausible that decreased glucose metabolism leads to an increased dependence on alternate energy substrates (e.g. ketones that arise from the oxidation of fat that is mobilized from storage [53]) to fuel survival. It is also feasible that defects in the capacity for neurons to use glucose as an energy substrate may lead to metabolic deficits that underpin the death of neurons in ALS. Indeed, decreased production of energy in the form of ATP and decreased glycolytic capacity in response to oxidative stress in NSC-34 motoneuron-like cells harbouring the SOD1^G93A^ mutation [114] indicate that impaired neuronal bioenergetics may play a role in the death of neurons in ALS.

## The metabolic demands of the neuron and the consequences of neuronal ATP depletion

The central nervous system comprises a complex network of highly organized and distinct neural circuits that mediate interneuronal communication. Energy demand in the brain is high. While accounting for approximately 2 % of total body mass, the human brain consumes 20 % of the total oxygen used by the body. Of the neural cell subtypes in the brain, energy consumption is predominantly demanded by the neurons, with astrocytes contributing only 5–15 % of the brain energy requirement [115].

Neurons are particularly active cells and, thus, have high metabolic demand. The metabolic processes in the neuron consists of (1) submembrane glycolysis, which is linked to the pumping of ions across the cell membrane, (2) aerobic glycolysis, which allows for the generation of pyruvate to fuel aerobic metabolism, and (3) the production of NADH/ATP in the mitochondria by means of the TCA cycle [116]. Although a low level of basal metabolism is critical for maintaining the survival of the cell, for active neurons, an increase in the metabolic demand that is required for the generation of action potentials [117] and their large surface area amounts to a considerable metabolic load that must be met through the generation of ATP. Neurons are extremely dependent on aerobic metabolism and oxygen use, but despite a large reservoir of ATP, reduced glycolytic and/or mitochondrial function modifies ATP availability, and glucose and oxygen deprivation in neurons results in cell death [118, 119]. Thus, when neurons are more active, increased local blood flow and increased substrate delivery from neighbouring cells is of critical importance to meet metabolic requirements and sustain cellular survival. As such, upon activation, neurons indirectly regulate their own metabolism by releasing by-products (e.g. nitric oxide and glutamate) that influence the surrounding cells and blood vessels [120–123]. This leads to the activation of astrocytes and increased levels of oxygen, lactate and glucose [117, 124, 125]. Despite categorical evidence that glucose is the chief energy substrate that is used by the brain to sustain metabolic demand, there is evidence to suggest that lactate can also be taken up by neurons to fuel aerobic metabolism [116, 124, 125]; it is postulated that the neuron-astrocyte lactate shuttle is the structure that permits the transfer of lactate from astrocytes to neurons for use as an additional metabolic substrate to fuel synaptic transmission [126–130]. With lactate being proposed to be a critical source of energy for active neurons, the lactate shuttle hypothesis postulates that neuronal-glutamate released during synaptic transmission drives aerobic glycolysis in astrocytes. Following this, glutamate is re-sequestered into astrocytes, resulting in the activation of the Na+/K+-ATPase, which in turn drives the use of cellular ATP. This initiates the uptake and processing of glucose and, finally, the release of lactate from astrocytes [127–129]. In line with a role for the lactate shuttle in the maintenance of neuronal energy demand, neurons express lactate dehydrogenase isoforms that favour the conversion of lactate to pyruvate and monocarboxylate transport (MCT2) receptors that take up pyruvate and lactate at high affinity. Thus, neurons in general, appear appropriately equipped to accommodate for their high metabolic demand [121, 131–134].

In ALS however, a combination of defective energy metabolism [14, 46], decreased glucose use in the cortex and spinal cord [49, 71, 135, 136], reduced expression of TCA cycle intermediates in the brain and spinal cord [137], damaged neuronal mitochondria [138–140], and mitochondrial electron transport chain dysfunction [8, 141, 142] suggest that a bioenergetic limitation exists throughout the course of disease and that the generation of neuronal ATP is compromised. Moreover, reduced lactate transport to neurons [119] and impaired lactate metabolism and impaired trafficking of lactate between neurons and astrocytes in SOD1-related ALS [143] suggest that defects in the lactate shuttle might further contribute to bioenergetic deficit in neuronal cells in ALS. Consequently, defects in neuronal metabolism may exist regardless of the provision of alternative energy substrates (e.g. through high calorie feeding [14, 50, 53, 62, 144] or ketogenic diet [145]) to sustain or improve neuronal energy supply. In this regard, treatments that serve to promote or recover the capacity to sustain cellular energy production will be fundamental to prevent neuronal death since deficits in the production of ATP in the presence of escalating metabolic pressures may underlie the selective and unrelenting death of neurons while exacerbating disease progression during later stages of ALS. Indeed, the consequences of ATP deficit has recently been highlighted in a modelling study that links cellular activity and vulnerability to degeneration to inadequate levels of cellular energy [146]. In this model, a deficit in ATP underpins higher metabolic cost to the neuron. This exacerbates energy deficit and disrupts cellular ionic gradients, triggering chronic and irreversible depolarisation (hyperexcitability) and neuronal death via ATP depletion [146].

Neuronal hyperexcitability is observed in ALS [11] and can be defined as an exaggerated response to a stimulus, which under normal circumstances would elicit an otherwise standard response. A positive correlation has been observed between increased axonal hyperexcitability [147–149] and disease progression in ALS patients [148], suggesting that alterations in the membrane excitability of axons that are distal to the neuron cell body might be central to the disease process. Critically, however, neuronal hyperexcitability, which may underpin the degeneration of neurons and their associated connections in ALS, has been found to occur early in the course of human ALS [11, 150] and in motor cortex layer V pyramidal neurons of SOD1^G93A^ mice [151].

The excitability of a neuron and the generation of action potentials within neurons are dependent upon calcium (Ca^2+^), sodium (Na^+^), and potassium (K^+^) channels. Importantly, the opening of voltage-gated K^+^ channels evokes the repolarisation of the cell to the resting potential. This allows the neuron to reduce calcium influx and thus decrease synaptic release of glutamate [152]. In light of a mathematical model proposing that axonal hyperexcitability in ALS might be due to impaired voltage-gated K^+^ currents [148], it has recently been shown that a similar impairment in voltage-gated K^+^ currents exists at the level of the neuron. Retigabine-induced activation of voltage-gated M-type K^+^ channels in SOD1 motor neurons derived from ALS patient-induced pluripotent stem cells (iPSCs) resulted in the reversal of intrinsic membrane hyperexcitability [12]. With evidence demonstrating that retigabine is also able to extend the survival of iPSC-derived SOD1 motor neurons from ALS patients, it is plausible that the activation of other K^+^ ion channels that function to attenuate neuronal depolarisation might produce protective effects in ALS. From an energetic perspective, increased Na^+^ influx associated with hyperexcitability in ALS may lead to overloading of the neuronal Na^+^-K^+^ ATPase-dependent pump resulting in excessive use of cellular ATP, energy failure, and neuronal death. Thus, other potential target candidates for attenuating chronic neuronal depolarisation in ALS may include K^+^ channels which couple the metabolic state of the cell to its activity.

## ATP-sensitive potassium (K_ATP_) channels: a new target in ALS?

K_ATP_ channels are octameric protein complexes that are made up of four pore-forming Kir6 inwardly rectifying potassium channel family (Kir) subunits and four regulatory sulfonylurea receptor (SUR) subunits [153, 154]. K_ATP_ channels play fundamental roles in cellular physiology. By regulating the flux of K^+^ across the cell membrane, K_ATP_ channels link the metabolic state of the cell to its electrical activity [155]. An increase in energy metabolism (and high ATP levels) drives the closure of K_ATP_ channels, resulting in membrane depolarization and electrical activity. By contrast, in response to metabolic deficit (and low ATP levels), K_ATP_ channels open, thereby driving a suppression in electrical activity [156]. Essentially glucosensing, K_ATP_ channels are regulated by the bioenergetic state of the cell (i.e. intracellular levels of ATP) [156]. Interestingly, K_ATP_ channels are also lactate sensing [157–160], and it has been postulated that they may modulate neuronal excitability in response to an increase in cytosolic ATP that is generated from the oxidation of astrocyte-derived lactate [161].

With mounting evidence to suggest that decreased cellular ATP production and subsequent alterations in cellular membrane excitability is associated with neurodegenerative disease and neuronal death [146, 162], it has been proposed that pharmacological mediators of K_ATP_ channels may prove to be promising targets for alleviating the neurodegenerative processes associated with disease or with neurotoxic insults [162–164]. However, while K_ATP_ channels are widely expressed [165–169], the biophysical, pharmacological, and metabolic properties of functional K_ATP_ channels are dictated by subunit composition [170, 171]. For example, channels that are formed by Kir6.2 and SUR1 are highly sensitive to diazoxide and are inhibited by ATP, and they express biophysical properties that are seen in pancreatic β cells [172, 173]. Conversely, Kir6.2/SUR2A K_ATP_ channels are somewhat insensitive to diazoxide, and they are predominantly expressed in the cardiac and skeletal muscle [171, 174]. Kir6.1/SUR2B or Kir6.2/SUR2B K_ATP_ channels possess properties reminiscent of those studied in the smooth muscle. While functional mitochondrial K_ATP_ channels have been proposed to be composed of various subunits [175–177], it is generally accepted that the molecular identity of such channels is yet to be determined. Of interest to neurodegeneration and ALS, Kir6.2/SUR1 K_ATP_ channels are widely expressed on neurons in the brain [168, 169, 178], and pharmacological targeting of such channels has proven to be promising in conditions that are associated with neuronal death.

Diazoxide is a well-known small molecule that activates K_ATP_ channels, including Kir6.2/SUR1 K_ATP_ channels [179]. The neuroprotective effects of diazoxide have been demonstrated in numerous studies. In cerebral ischemia-reperfusion injury, diazoxide reduces levels of reactive oxygen species, decreases DNA oxidative damage, inhibits apoptosis [180–182], and reduces infarct size during ischemia [183]. In the context of Parkinson’s disease, diazoxide reduces akinesia [184], protects dopaminergic neurons from death by reducing astrocyte and microglial activation [185], and reduces neuroinflammation associated with activated microglia [186]. In in vitro and in vivo models of Alzheimer’s disease, activation of K_ATP_ channels by diazoxide protects against β-amyloid toxicity, reducing protein aggregation and tau hyperphosphorylation [163, 187]. Finally, in addition to having been shown to reduce glutamate excitotoxicity in epilepsy [188], diazoxide also protects NSC-34 motoneurons from glutamate-mediated cell death, hydrogen peroxide-mediated cell death, and inflammatory damage associated with microglial activation, while decreasing neuronal death in hippocampal slices after N-methyl-D-aspartic acid (NMDA)-induced excitotoxicity [189].

The use of diazoxide and the investigation of its neuroprotective potential and role in ALS however is relatively less well studied. Interestingly, however, a patent describing oral administration of low doses of diazoxide in SOD1^G93A^ mice reported improved median values for survival when compared to non-diazoxide-supplemented SOD1^G93A^ mice [190]. Whether this improved survival outcome in diazoxide-supplemented mice is due to the ability for diazoxide to (a) improve insulin sensitivity and glucose metabolism (thereby presumably counteracting systemic defects in metabolic homeostasis [191]), (b) cross the blood-brain barrier [192] to counteract intrinsic cellular excitability (as has been shown in immature entorhinal cortex neurons [193]), or (c) counteract chronic depolarization that might arise from persistent ATP deficit [146] in response to decreased glucose use [49, 71, 135, 136] and defective function of the astrocyte-lactate shuttle in ALS [143] remains to be determined. Regardless, there is substantial evidence to suggest that the pharmacological modulation of metabolically sensitive K_ATP_ channels by diazoxide (or other specific activators) represents a promising approach for obtaining neuroprotection in neurodegenerative diseases, including ALS.

## Conclusions and considerations

**Fig. 2:**
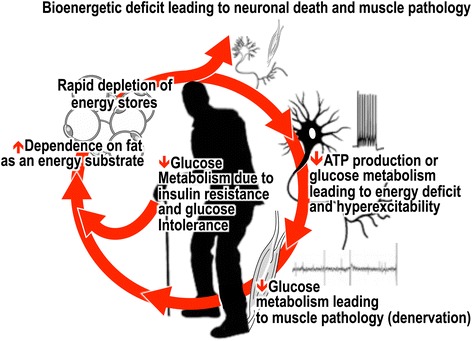
Decreased production of adenosine triphosphate or decreased glucose metabolism in neurons and decreased glucose metabolism in the skeletal muscle may contribute to the hyperexcitability and selective degeneration of upper and lower motor neurons and muscle pathology/denervation in ALS, respectively. Insulin resistance and glucose intolerance may underpin an inability to efficiently use glucose as an energy substrate. Overall, an inabillity to use glucose in the periphery, in neurons and in skeletal muscle will result in an increased dependence on the use of fat as an energy substrate to offset energy deficit. With escalating metabolic pressure, the rapid depletion of endogenous energy stores will result in a catastrophic failure to meet increased metabolic demand. Thus, a vicious cycle of bioenergetic deficit may underpin or exacerbate disease pathogenesis in ALS

The debilitating nature of ALS and the lack of effective treatments against this insidious disease highlight the need to identify therapeutic targets that are amenable to therapy. While systemic manifestation of energy deficit presenting as hypermetabolism, malnutrition, and decreased fat stores (due to increased dependence on fat as an energy substrate) is clearly associated with disease course, what is more striking is the notion that bioenergetic deficit (due to decreased ATP production or decreased glucose metabolism) may contribute in part to the hyperexcitability and selective degeneration of upper and lower motor neurons and muscle pathology/denervation in ALS. When considering all metabolic components, it may well be that a vicious cycle of bioenergetic deficit underpins or exacerbates disease pathogenesis in ALS (Fig. 2). Whether the activation or deactivation of metabolically sensitive K_ATP_ channels and their regulation of systemic metabolic homeostasis and cellular excitability ultimately contribute to neuronal hyperexcitability and the subsequent degeneration of neural networks that are linked to hyperexcitable cells in ALS remains unknown. Nonetheless, the potential for K_ATP_ channels to be novel targets for the treatment of ALS is of significance, as the current availability of a number of compounds that are selective for K_ATP_ channels will greatly facilitate the pharmacological modulation of K_ATP_ channels as an avenue for future scientific investigation in ALS. The knowledge that promises to be gained from such studies will determine whether targeting of metabolic pathways or accommodation for metabolic dysfunction presents as promising therapeutic targets in ALS.

## Abbreviations

ALS: amyotrophic lateral sclerosis
ATP: adenosine triphosphate
BMI: body mass index
Ca^2+^: calcium
IGF-1: insulin-like growth factor 1
iPSCs: induced pluripotent stem cells
K^+^: potassium
Kir: inwardly rectifying potassium channels
MCT2: monocarboxylate transporter 2
Na^+^: sodium
NADH: nicotinamide adenine dinucleotide (reduced)
NMDA: N-methyl-D-aspartic acid
SOD1: superoxide dismutase 1
SUR: sulfonylurea
TCA: tricarboxylic acid

## References

[1] Charcot JM, Joffory A (1869). Deux cas d’atrophie musculaire progressive avec lesions de la substance grise et des faisceaux antero-lateraux de la moelle epiniere. Arch Physiol Neurol Pathol.

[2] Kiernan MC, Vucic S, Cheah BC, Turner MR, Eisen A, Hardiman O (2011). Amyotrophic lateral sclerosis. Lancet.

[3] Blair IP, Williams KL, Warraich ST, Durnall JC, Thoeng AD, Manavis J (2009). FUS mutations in amyotrophic lateral sclerosis: clinical, pathological, neurophysiological and genetic analysis. J Neurol Neurosurg Psychiatry.

[4] DeJesus-Hernandez M, Mackenzie IR, Boeve BF, Boxer AL, Baker M, Rutherford NJ (2011). Expanded GGGGCC hexanucleotide repeat in noncoding region of C9ORF72 causes chromosome 9p-linked FTD and ALS. Neuron.

[5] Kabashi E, Valdmanis PN, Dion P, Spiegelman D, McConkey BJ, Vande Velde C (2008). TARDBP mutations in individuals with sporadic and familial amyotrophic lateral sclerosis. Nat Genet.

[6] Maruyama H, Morino H, Ito H, Izumi Y, Kato H, Watanabe Y (2010). Mutations of optineurin in amyotrophic lateral sclerosis. Nature.

[7] Renton AE, Majounie E, Waite A, Simon-Sanchez J, Rollinson S, Gibbs JR (2011). A hexanucleotide repeat expansion in C9ORF72 is the cause of chromosome 9p21-linked ALS-FTD. Neuron.

[8] Crugnola V, Lamperti C, Lucchini V, Ronchi D, Peverelli L, Prelle A (2010). Mitochondrial respiratory chain dysfunction in muscle from patients with amyotrophic lateral sclerosis. Arch Neurol.

[9] Haidet-Phillips AM, Hester ME, Miranda CJ, Meyer K, Braun L, Frakes A (2011). Astrocytes from familial and sporadic ALS patients are toxic to motor neurons. Nat Biotechnol.

[10] Yamanaka K, Chun SJ, Boillee S, Fujimori-Tonou N, Yamashita H, Gutmann DH (2008). Astrocytes as determinants of disease progression in inherited amyotrophic lateral sclerosis. Nat Neurosci.

[11] Vucic S, Nicholson GA, Kiernan MC (2008). Cortical hyperexcitability may precede the onset of familial amyotrophic lateral sclerosis. Brain.

[12] Wainger BJ, Kiskinis E, Mellin C, Wiskow O, Han SS, Sandoe J (2014). Intrinsic membrane hyperexcitability of amyotrophic lateral sclerosis patient-derived motor neurons. Cell Rep.

[13] Shaw PJ, Ince PG (1997). Glutamate, excitotoxicity and amyotrophic lateral sclerosis. J Neurol.

[14] Dupuis L, Oudart H, Rene F, Gonzalez de Aguilar JL, Loeffler JP (2004). Evidence for defective energy homeostasis in amyotrophic lateral sclerosis: benefit of a high-energy diet in a transgenic mouse model. Proc Natl Acad Sci U S A.

[15] Renton AE, Chio A, Traynor BJ (2014). State of play in amyotrophic lateral sclerosis genetics. Nat Neurosci.

[16] Rowland LP, Schneider LA (2001). Amyotrophic lateral sclerosis. N Engl J Med.

[17] Rosen DR, Siddique T, Patterson D, Figlewicz DA, Sapp P, Hentati A (1993). Mutations in Cu/Zn superoxide dismutase gene are associated with familial amyotrophic lateral sclerosis. Nature.

[18] Sreedharan J, Blair IP, Tripathi VB, Hu X, Vance C, Rogelj B (2008). TDP-43 mutations in familial and sporadic amyotrophic lateral sclerosis. Science.

[19] Kwiatkowski TJ, Bosco DA, Leclerc AL, Tamrazian E, Vanderburg CR, Russ C (2009). Mutations in the FUS/TLS gene on chromosome 16 cause familial amyotrophic lateral sclerosis. Science.

[20] Vance C, Rogelj B, Hortobagyi T, De Vos KJ, Nishimura AL, Sreedharan J (2009). Mutations in FUS, an RNA processing protein, cause familial amyotrophic lateral sclerosis type 6. Science.

[21] Al-Chalabi A, Calvo A, Chio A, Colville S, Ellis CM, Hardiman O (2014). Analysis of amyotrophic lateral sclerosis as a multistep process: a population-based modelling study. Lancet Neurol.

[22] Chio A, Benzi G, Dossena M, Mutani R, Mora G (2005). Severely increased risk of amyotrophic lateral sclerosis among Italian professional football players. Brain.

[23] Chio A, Calvo A, Dossena M, Ghiglione P, Mutani R, Mora G (2009). ALS in Italian professional soccer players: the risk is still present and could be soccer-specific. Amyotroph Lateral Scler.

[24] Scarmeas N, Shih T, Stern Y, Ottman R, Rowland LP (2002). Premorbid weight, body mass, and varsity athletics in ALS. Neurology.

[25] Masseret E, Banack S, Boumediene F, Abadie E, Brient L, Pernet F (2013). Investigation Dietary BMAA exposure in an amyotrophic lateral sclerosis cluster from southern France. PLoS One.

[26] Plato CC, Garruto RM, Galasko D, Craig UK, Plato M, Gamst A, et al. Amyotrophic lateral sclerosis and parkinsonism-dementia complex of Guam: changing incidence rates during the past 60 years. Am J Epidemiol. 2003;157:149–57. 10.1093/aje/kwf175 12522022

[27] Kamel F, Umbach DM, Bedlack RS, Richards M, Watson M, Alavanja MC (2012). Pesticide exposure and amyotrophic lateral sclerosis. Neurotoxicology.

[28] Malek AM, Barchowsky A, Bowser R, Youk A, Talbott EO (2012). Pesticide exposure as a risk factor for amyotrophic lateral sclerosis: a meta-analysis of epidemiological studies: pesticide exposure as a risk factor for ALS. Environ Res.

[29] Armon C (2003). An evidence-based medicine approach to the evaluation of the role of exogenous risk factors in sporadic amyotrophic lateral sclerosis. Neuroepidemiology.

[30] Armon C (2009). Smoking may be considered an established risk factor for sporadic ALS. Neurology.

[31] Ascherio A, Weisskopf MG, O’Reilly EJ, Jacobs EJ, McCullough ML, Calle EE (2005). Vitamin E intake and risk of amyotrophic lateral sclerosis. Ann Neurol.

[32] Fitzgerald KC, O’Reilly EJ, Falcone GJ, McCullough ML, Park Y, Kolonel LN (2014). Dietary omega-3 polyunsaturated fatty acid intake and risk for amyotrophic lateral sclerosis. JAMA Neurol.

[33] Okamoto K, Kihira T, Kondo T, Kobashi G, Washio M, Sasaki S (2007). Nutritional status and risk of amyotrophic lateral sclerosis in Japan. Amyotroph Lateral Scler.

[34] Veldink JH, Kalmijn S, Groeneveld GJ, Wunderink W, Koster A, de Vries JH (2007). Intake of polyunsaturated fatty acids and vitamin E reduces the risk of developing amyotrophic lateral sclerosis. J Neurol Neurosurg Psychiatry.

[35] Wang H, O’Reilly EJ, Weisskopf MG, Logroscino G, McCullough ML, Schatzkin A (2011). Vitamin E intake and risk of amyotrophic lateral sclerosis: a pooled analysis of data from 5 prospective cohort studies. Am J Epidemiol.

[36] Desport JC, Preux PM, Truong TC, Vallat JM, Sautereau D, Couratier P (1999). Nutritional status is a prognostic factor for survival in ALS patients. Neurology.

[37] Limousin N, Blasco H, Corcia P, Gordon PH, De Toffol B, Andres C (2010). Malnutrition at the time of diagnosis is associated with a shorter disease duration in ALS. J Neurol Sci.

[38] Gallo V, Wark PA, Jenab M, Pearce N, Brayne C, Vermeulen R (2013). Prediagnostic body fat and risk of death from amyotrophic lateral sclerosis: the EPIC cohort. Neurology.

[39] Jawaid A, Murthy SB, Wilson AM, Qureshi SU, Amro MJ, Wheaton M (2010). A decrease in body mass index is associated with faster progression of motor symptoms and shorter survival in ALS. Amyotroph Lateral Scler.

[40] O’Reilly EJ, Wang H, Weisskopf MG, Fitzgerald KC, Falcone G, McCullough ML (2013). Premorbid body mass index and risk of amyotrophic lateral sclerosis. Amyotroph Lateral Scler Frontotemporal Degener.

[41] Shimizu T, Nagaoka U, Nakayama Y, Kawata A, Kugimoto C, Kuroiwa Y (2012). Reduction rate of body mass index predicts prognosis for survival in amyotrophic lateral sclerosis: a multicenter study in Japan. Amyotroph Lateral Scler.

[42] Ingre C, Roos PM, Piehl F, Kamel F, Fang F (2015). Risk factors for amyotrophic lateral sclerosis. Clin Epidemiol.

[43] Reyes ET, Perurena OH, Festoff BW, Jorgensen R, Moore WV (1984). Insulin resistance in amyotrophic lateral sclerosis. J Neurol Sci.

[44] Van den Bergh R, Swerts L, Hendrikx A, Boni L, Meulepas E (1977). Adipose tissue cellularity in patients with amyotrophic lateral sclerosis. Clin Neurol Neurosurg.

[45] Cistaro A, Valentini MC, Chio A, Nobili F, Calvo A, Moglia C (2012). Brain hypermetabolism in amyotrophic lateral sclerosis: a FDG PET study in ALS of spinal and bulbar onset. Eur J Nucl Med Mol Imaging.

[46] Dalakas MC, Hatazawa J, Brooks RA, Di Chiro G (1987). Lowered cerebral glucose utilization in amyotrophic lateral sclerosis. Ann Neurol.

[47] Desport JC, Preux PM, Magy L, Boirie Y, Vallat JM, Beaufrere B (2001). Factors correlated with hypermetabolism in patients with amyotrophic lateral sclerosis. Am J Clin Nutr.

[48] Desport JC, Torny F, Lacoste M, Preux PM, Couratier P (2005). Hypermetabolism in ALS: correlations with clinical and paraclinical parameters. Neurodegener Dis.

[49] Dodge JC, Treleaven CM, Fidler JA, Tamsett TJ, Bao C, Searles M (2013). Metabolic signatures of amyotrophic lateral sclerosis reveal insights into disease pathogenesis. Proc Natl Acad Sci U S A.

[50] Dorst J, Cypionka J, Ludolph AC (2013). High-caloric food supplements in the treatment of amyotrophic lateral sclerosis: a prospective interventional study. Amyotroph Lateral Scler Frontotemporal Degener.

[51] Dorst J, Kuhnlein P, Hendrich C, Kassubek J, Sperfeld AD, Ludolph AC (2011). Patients with elevated triglyceride and cholesterol serum levels have a prolonged survival in amyotrophic lateral sclerosis. J Neurol.

[52] Dupuis L, Pradat PF, Ludolph AC, Loeffler JP (2011). Energy metabolism in amyotrophic lateral sclerosis. Lancet Neurol.

[53] Fergani A, Oudart H, Gonzalez De Aguilar JL, Fricker B, Rene F, Hocquette JF (2007). Increased peripheral lipid clearance in an animal model of amyotrophic lateral sclerosis. J Lipid Res.

[54] Funalot B, Desport JC, Sturtz F, Camu W, Couratier P (2009). High metabolic level in patients with familial amyotrophic lateral sclerosis. Amyotroph Lateral Scler.

[55] Genton L, Viatte V, Janssens JP, Heritier AC, Pichard C (2011). Nutritional state, energy intakes and energy expenditure of amyotrophic lateral sclerosis (ALS) patients. Clin Nutr.

[56] Jawaid A, Salamone AR, Strutt AM, Murthy SB, Wheaton M, McDowell EJ (2010). ALS disease onset may occur later in patients with pre-morbid diabetes mellitus. Eur J Neurol.

[57] Kasarskis EJ, Berryman S, Vanderleest JG, Schneider AR, McClain CJ (1996). Nutritional status of patients with amyotrophic lateral sclerosis: relation to the proximity of death. Am J Clin Nutr.

[58] Ngo ST, Steyn FJ, McCombe PA (2014). Body mass index and dietary intervention: implications for prognosis of amyotrophic lateral sclerosis. J Neurol Sci.

[59] Paganoni S, Deng J, Jaffa M, Cudkowicz ME, Wills AM (2011). Body mass index, not dyslipidemia, is an independent predictor of survival in amyotrophic lateral sclerosis. Muscle Nerve.

[60] Palamiuc L, Schlagowski A, Ngo ST, Vernay A, Grosch S, Henriques A (2015). A metabolic switch towards lipid use in glycolytic muscle is an early pathologic event in a mouse model of amyotrophic lateral sclerosis. EMBO Mol Med.

[61] Pradat PF, Bruneteau G, Gordon PH, Dupuis L, Bonnefont-Rousselot D, Simon D (2010). Impaired glucose tolerance in patients with amyotrophic lateral sclerosis. Amyotroph Lateral Scler.

[62] Wills AM, Hubbard J, Macklin EA, Glass J, Tandan R, Simpson EP (2014). Hypercaloric enteral nutrition in patients with amyotrophic lateral sclerosis: a randomised, double-blind, placebo-controlled phase 2 trial. Lancet.

[63] Greenwood DI (2013). Nutrition management of amyotrophic lateral sclerosis. Nutr Clin Pract.

[64] Schwartz MW, Woods SC, Porte D, Seeley RJ, Baskin DG (2000). Central nervous system control of food intake. Nature.

[65] Meier U, Gressner AM (2004). Endocrine regulation of energy metabolism: review of pathobiochemical and clinical chemical aspects of leptin, ghrelin, adiponectin, and resistin. Clin Chem.

[66] Moller N, Jorgensen JO (2009). Effects of growth hormone on glucose, lipid, and protein metabolism in human subjects. Endocr Rev.

[67] Steyn FJ. Nutrient sensing overrides somatostatin and GHRH to control pulsatile GH release. J Neuroendocrinol. 2015. 10.1111/jne.12278 25808924

[68] Morselli LL, Bongioanni P, Genovesi M, Licitra R, Rossi B, Murri L (2006). Growth hormone secretion is impaired in amyotrophic lateral sclerosis. Clin Endocrinol.

[69] Dupuis L, Corcia P, Fergani A, Gonzalez De Aguilar JL, Bonnefont-Rousselot D, Bittar R (2008). Dyslipidemia is a protective factor in amyotrophic lateral sclerosis. Neurology.

[70] Cistaro A, Pagani M, Montuschi A, Calvo A, Moglia C, Canosa A (2014). The metabolic signature of C9ORF72-related ALS: FDG PET comparison with nonmutated patients. Eur J Nucl Med Mol Imaging.

[71] Hatazawa J, Brooks RA, Dalakas MC, Mansi L, Di Chiro G (1988). Cortical motor-sensory hypometabolism in amyotrophic lateral sclerosis: a PET study. J Comput Assist Tomogr.

[72] Pagani M, Chio A, Valentini MC, Oberg J, Nobili F, Calvo A (2014). Functional pattern of brain FDG-PET in amyotrophic lateral sclerosis. Neurology.

[73] Chiang PM, Ling J, Jeong YH, Price DL, Aja SM, Wong PC (2010). Deletion of TDP-43 down-regulates Tbc1d1, a gene linked to obesity, and alters body fat metabolism. Proc Natl Acad Sci U S A.

[74] Stallings NR, Puttaparthi K, Dowling KJ, Luther CM, Burns DK, Davis K (2013). TDP-43, an ALS linked protein, regulates fat deposition and glucose homeostasis. PLoS One.

[75] Shan X, Chiang PM, Price DL, Wong PC (2010). Altered distributions of Gemini of coiled bodies and mitochondria in motor neurons of TDP-43 transgenic mice. Proc Natl Acad Sci U S A.

[76] Xu YF, Gendron TF, Zhang YJ, Lin WL, D’Alton S, Sheng H (2010). Wild-type human TDP-43 expression causes TDP-43 phosphorylation, mitochondrial aggregation, motor deficits, and early mortality in transgenic mice. J Neurosci.

[77] Wang T, Jiang X, Chen G, Xu J (2015). Interaction of amyotrophic lateral sclerosis/frontotemporal lobar degeneration-associated fused-in-sarcoma with proteins involved in metabolic and protein degradation pathways. Neurobiol Aging.

[78] Watt MJ, Spriet LL (2010). Triacylglycerol lipases and metabolic control: implications for health and disease. Am J Physiol Endocrinol Metab.

[79] Ngo ST, Steyn FJ, Huang L, Mantovani S, Pfluger CMM, Woodruff TM, O’Sullivan JD, Henderson RD, McCombe PA. Altered expression of metabolic proteins and adipokines in patients with amyotrophic lateral sclerosis. J Neurol Sci 2015; doi:10.1016/j.jns.2015.06.053 10.1016/j.jns.2015.06.053 26198021

[80] Henriques A, Blasco H, Fleury MC, Corcia P, Echaniz-Laguna A, Robelin L (2015). Blood cell palmitoleate-palmitate ratio is an independent prognostic factor for amyotrophic lateral sclerosis. PLoS One.

[81] Lindauer E, Dupuis L, Muller HP, Neumann H, Ludolph AC, Kassubek J (2013). Adipose tissue distribution predicts survival in amyotrophic lateral sclerosis. PLoS One.

[82] Bechmann LP, Hannivoort RA, Gerken G, Hotamisligil GS, Trauner M, Canbay A (2012). The interaction of hepatic lipid and glucose metabolism in liver diseases. J Hepatol.

[83] Araki H (1978). Hepatic ultrastructural study in amyotrophic lateral sclerosis (ALS). No to shinkei =. Brain Nerve.

[84] Hirayama K (1982). Electron microscopic, abnormalities of the liver in amyotrophic lateral sclerosis. No to shinkei =. Brain Nerve.

[85] Masui Y, Mozai T, Kakehi K (1985). Functional and morphometric study of the liver in motor neuron disease. J Neurol.

[86] Nakano Y, Hirayama K, Terao K (1987). Hepatic ultrastructural changes and liver dysfunction in amyotrophic lateral sclerosis. Arch Neurol.

[87] Nodera H, Takamatsu N, Muguruma N, Ukimoto K, Nishio S, Oda M (2015). Frequent hepatic steatosis in amyotrophic lateral sclerosis: implication for systemic involvement. Neurol Clin Neurosci.

[88] Finkelstein A, Kunis G, Seksenyan A, Ronen A, Berkutzki T, Azoulay D (2011). Abnormal changes in NKT cells, the IGF-1 axis, and liver pathology in an animal model of ALS. PLoS One.

[89] DeFronzo RA, Jacot E, Jequier E, Maeder E, Wahren J, Felber JP (1981). The effect of insulin on the disposal of intravenous glucose. Results from indirect calorimetry and hepatic and femoral venous catheterization. Diabetes.

[90] Baron AD, Brechtel G, Wallace P, Edelman SV (1988). Rates and tissue sites of non-insulin- and insulin-mediated glucose uptake in humans. Am J Physiol.

[91] Dobrowolny G, Aucello M, Rizzuto E, Beccafico S, Mammucari C, Boncompagni S (2008). Skeletal muscle is a primary target of SOD1G93A-mediated toxicity. Cell Metab.

[92] Wong M, Martin LJ (2010). Skeletal muscle-restricted expression of human SOD1 causes motor neuron degeneration in transgenic mice. Hum Mol Genet.

[93] Dupuis L, Gonzalez de Aguilar JL, Echaniz-Laguna A, Eschbach J, Rene F, Oudart H (2009). Muscle mitochondrial uncoupling dismantles neuromuscular junction and triggers distal degeneration of motor neurons. PLoS One.

[94] Russell AP, Wada S, Vergani L, Hock MB, Lamon S, Leger B (2013). Disruption of skeletal muscle mitochondrial network genes and miRNAs in amyotrophic lateral sclerosis. Neurobiol Dis.

[95] Vielhaber S, Winkler K, Kirches E, Kunz D, Buchner M, Feistner H (1999). Visualization of defective mitochondrial function in skeletal muscle fibers of patients with sporadic amyotrophic lateral sclerosis. J Neurol Sci.

[96] Wiedemann FR, Winkler K, Kuznetsov AV, Bartels C, Vielhaber S, Feistner H (1998). Impairment of mitochondrial function in skeletal muscle of patients with amyotrophic lateral sclerosis. J Neurol Sci.

[97] Derave W, Van Den Bosch L, Lemmens G, Eijnde BO, Robberecht W, Hespel P (2003). Skeletal muscle properties in a transgenic mouse model for amyotrophic lateral sclerosis: effects of creatine treatment. Neurobiol Dis.

[98] Siciliano G, D’Avino C, Del Corona A, Barsacchi R, Kusmic C, Rocchi A (2002). Impaired oxidative metabolism and lipid peroxidation in exercising muscle from ALS patients. Amyotroph Lateral Scler Other Motor Neuron Disord.

[99] Siciliano G, Pastorini E, Pasquali L, Manca ML, Iudice A, Murri L (2001). Impaired oxidative metabolism in exercising muscle from ALS patients. J Neurol Sci.

[100] Smittkamp SE, Morris JK, Bomhoff GL, Chertoff ME, Geiger PC, Stanford JA (2014). SOD1-G93A mice exhibit muscle-fiber-type-specific decreases in glucose uptake in the absence of whole-body changes in metabolism. Neurodegener Dis.

[101] Echaniz-Laguna A, Zoll J, Ponsot E, N’Guessan B, Tranchant C, Loeffler JP (2006). Muscular mitochondrial function in amyotrophic lateral sclerosis is progressively altered as the disease develops: a temporal study in man. Exp Neurol.

[102] Rai M, Nongthomba U, Grounds MD (2014). Skeletal muscle degeneration and regeneration in mice and flies. Curr Top Dev Biol.

[103] Deng HX, Shi Y, Furukawa Y, Zhai H, Fu R, Liu E (2006). Conversion to the amyotrophic lateral sclerosis phenotype is associated with intermolecular linked insoluble aggregates of SOD1 in mitochondria. Proc Natl Acad Sci U S A.

[104] Graffmo KS, Forsberg K, Bergh J, Birve A, Zetterstrom P, Andersen PM (2013). Expression of wild-type human superoxide dismutase-1 in mice causes amyotrophic lateral sclerosis. Hum Mol Genet.

[105] Wang L, Deng HX, Grisotti G, Zhai H, Siddique T, Roos RP (2009). Wild-type SOD1 overexpression accelerates disease onset of a G85R SOD1 mouse. Hum Mol Genet.

[106] Witan H, Kern A, Koziollek-Drechsler I, Wade R, Behl C, Clement AM (2008). Heterodimer formation of wild-type and amyotrophic lateral sclerosis-causing mutant Cu/Zn-superoxide dismutase induces toxicity independent of protein aggregation. Hum Mol Genet.

[107] Watt MJ, Hoy AJ (2012). Lipid metabolism in skeletal muscle: generation of adaptive and maladaptive intracellular signals for cellular function. Am J Physiol Endocrinol Metab.

[108] Laffel L (1999). Ketone bodies: a review of physiology, pathophysiology and application of monitoring to diabetes. Diabetes Metab Res Rev.

[109] Gibbons CJ, Thornton EW, Young CA (2013). The patient experience of fatigue in motor neurone disease. Front Psychol.

[110] Gallardo G, Barowski J, Ravits J, Siddique T, Lingrel JB, Robertson J (2014). An alpha2-Na/K ATPase/alpha-adducin complex in astrocytes triggers non-cell autonomous neurodegeneration. Nat Neurosci.

[111] Renard D, Collombier L, Castelnovo G, Fourcade G, Kotzki PO, LaBauge P (2011). Brain FDG-PET changes in ALS and ALS-FTD. Acta Neurol Belg.

[112] Van Laere K, Vanhee A, Verschueren J, De Coster L, Driesen A, Dupont P (2014). Value of 18fluorodeoxyglucose-positron-emission tomography in amyotrophic lateral sclerosis: a prospective study. JAMA Neurol.

[113] Miyazaki K, Masamoto K, Morimoto N, Kurata T, Mimoto T, Obata T (2012). Early and progressive impairment of spinal blood flow-glucose metabolism coupling in motor neuron degeneration of ALS model mice. J Cereb Blood Flow Metab.

[114] Richardson K, Allen SP, Mortiboys H, Grierson AJ, Wharton SB, Ince PG (2013). The effect of SOD1 mutation on cellular bioenergetic profile and viability in response to oxidative stress and influence of mutation-type. PLoS One.

[115] Attwell D, Laughlin SB (2001). An energy budget for signaling in the grey matter of the brain. J Cereb Blood Flow Metab.

[116] Aubert A, Pellerin L, Magistretti PJ, Costalat R (2007). A coherent neurobiological framework for functional neuroimaging provided by a model integrating compartmentalized energy metabolism. Proc Natl Acad Sci U S A.

[117] Sokoloff L (1999). Energetics of functional activation in neural tissues. Neurochem Res.

[118] Goldberg MP, Choi DW (1993). Combined oxygen and glucose deprivation in cortical cell culture: calcium-dependent and calcium-independent mechanisms of neuronal injury. J Neurosci.

[119] Lee Y, Morrison BM, Li Y, Lengacher S, Farah MH, Hoffman PN (2012). Oligodendroglia metabolically support axons and contribute to neurodegeneration. Nature.

[120] Hertz L, Peng L, Dienel GA (2007). Energy metabolism in astrocytes: high rate of oxidative metabolism and spatiotemporal dependence on glycolysis/glycogenolysis. J Cereb Blood Flow Metab.

[121] Tsacopoulos M, Magistretti PJ (1996). Metabolic coupling between glia and neurons. J Neurosci.

[122] Dienel GA, Hertz L (2001). Glucose and lactate metabolism during brain activation. J Neurosci Res.

[123] Gordon GR, Choi HB, Rungta RL, Ellis-Davies GC, MacVicar BA (2008). Brain metabolism dictates the polarity of astrocyte control over arterioles. Nature.

[124] Galeffi F, Foster KA, Sadgrove MP, Beaver CJ, Turner DA (2007). Lactate uptake contributes to the NAD(P)H biphasic response and tissue oxygen response during synaptic stimulation in area CA1 of rat hippocampal slices. J Neurochem.

[125] Hu Y, Wilson GS (1997). A temporary local energy pool coupled to neuronal activity: fluctuations of extracellular lactate levels in rat brain monitored with rapid-response enzyme-based sensor. J Neurochem.

[126] Guzman M, Blazquez C (2001). Is there an astrocyte-neuron ketone body shuttle?. Trends Endocrinol Metab.

[127] Pellerin L, Magistretti PJ (1994). Glutamate uptake into astrocytes stimulates aerobic glycolysis: a mechanism coupling neuronal activity to glucose utilization. Proc Natl Acad Sci U S A.

[128] Pellerin L, Pellegri G, Bittar PG, Charnay Y, Bouras C, Martin JL (1998). Evidence supporting the existence of an activity-dependent astrocyte-neuron lactate shuttle. Dev Neurosci.

[129] Pellerin L, Bouzier-Sore AK, Aubert A, Serres S, Merle M, Costalat R (2007). Activity-dependent regulation of energy metabolism by astrocytes: an update. Glia.

[130] Tarczyluk MA, Nagel DA, O’Neil JD, Parri HR, Tse EH, Coleman MD (2013). Functional astrocyte-neuron lactate shuttle in a human stem cell-derived neuronal network. J Cereb Blood Flow Metab.

[131] Hertz L, Dienel GA (2005). Lactate transport and transporters: general principles and functional roles in brain cells. J Neurosci Res.

[132] Verkhratsky A, Toescu EC (2006). Neuronal-glial networks as substrate for CNS integration. J Cell Mol Med.

[133] Bittar PG, Charnay Y, Pellerin L, Bouras C, Magistretti PJ (1996). Selective distribution of lactate dehydrogenase isoenzymes in neurons and astrocytes of human brain. J Cereb Blood Flow Metab.

[134] O’Brien J, Kla KM, Hopkins IB, Malecki EA, McKenna MC (2007). Kinetic parameters and lactate dehydrogenase isozyme activities support possible lactate utilization by neurons. Neurochem Res.

[135] Browne SE, Yang L, DiMauro JP, Fuller SW, Licata SC, Beal MF (2006). Bioenergetic abnormalities in discrete cerebral motor pathways presage spinal cord pathology in the G93A SOD1 mouse model of ALS. Neurobiol Dis.

[136] Ludolph AC, Langen KJ, Regard M, Herzog H, Kemper B, Kuwert T (1992). Frontal lobe function in amyotrophic lateral sclerosis: a neuropsychologic and positron emission tomography study. Acta Neurol Scand.

[137] Niessen HG, Debska-Vielhaber G, Sander K, Angenstein F, Ludolph AC, Hilfert L (2007). Metabolic progression markers of neurodegeneration in the transgenic G93A-SOD1 mouse model of amyotrophic lateral sclerosis. Eur J Neurosci.

[138] Magrane J, Cortez C, Gan WB, Manfredi G (2014). Abnormal mitochondrial transport and morphology are common pathological denominators in SOD1 and TDP43 ALS mouse models. Hum Mol Genet.

[139] Stribl C, Samara A, Trumbach D, Peis R, Neumann M, Fuchs H (2014). Mitochondrial dysfunction and decrease in body weight of a transgenic knock-in mouse model for TDP-43. J Biol Chem.

[140] Carri MT, Valle C, Bozzo F, Cozzolino M (2015). Oxidative stress and mitochondrial damage: importance in non-SOD1 ALS. Front Cell Neurosci.

[141] Jung C, Higgins CM, Xu Z (2002). Mitochondrial electron transport chain complex dysfunction in a transgenic mouse model for amyotrophic lateral sclerosis. J Neurochem.

[142] Wiedemann FR, Manfredi G, Mawrin C, Beal MF, Schon EA (2002). Mitochondrial DNA and respiratory chain function in spinal cords of ALS patients. J Neurochem.

[143] Ferraiuolo L, Higginbottom A, Heath PR, Barber S, Greenald D, Kirby J (2011). Dysregulation of astrocyte-motoneuron cross-talk in mutant superoxide dismutase 1-related amyotrophic lateral sclerosis. Brain.

[144] Mattson MP, Cutler RG, Camandola S (2007). Energy intake and amyotrophic lateral sclerosis. Neuromolecular Med.

[145] Zhao Z, Lange DJ, Voustianiouk A, MacGrogan D, Ho L, Suh J (2006). A ketogenic diet as a potential novel therapeutic intervention in amyotrophic lateral sclerosis. BMC Neurosci.

[146] Le Masson G, Przedborski S, Abbott LF (2014). A computational model of motor neuron degeneration. Neuron.

[147] Bostock H, Sharief MK, Reid G, Murray NM (1995). Axonal ion channel dysfunction in amyotrophic lateral sclerosis. Brain.

[148] Kanai K, Kuwabara S, Misawa S, Tamura N, Ogawara K, Nakata M (2006). Altered axonal excitability properties in amyotrophic lateral sclerosis: impaired potassium channel function related to disease stage. Brain.

[149] Nakata M, Kuwabara S, Kanai K, Misawa S, Tamura N, Sawai S (2006). Distal excitability changes in motor axons in amyotrophic lateral sclerosis. Clin Neurophysiol.

[150] Vucic S, Kiernan MC (2006). Novel threshold tracking techniques suggest that cortical hyperexcitability is an early feature of motor neuron disease. Brain.

[151] Fogarty MJ, Noakes PG, Bellingham MC (2015). Motor cortex layer V pyramidal neurons exhibit dendritic regression, spine loss, and increased synaptic excitation in the presymptomatic hSOD1G93A mouse model of amyotrophic lateral sclerosis. J Neurosci.

[152] Lawson K, McKay NG (2006). Modulation of potassium channels as a therapeutic approach. Curr Pharm Des.

[153] Clement JP, Kunjilwar K, Gonzalez G, Schwanstecher M, Panten U, Aguilar-Bryan L (1997). Association and stoichiometry of K(ATP) channel subunits. Neuron.

[154] Shyng S, Nichols CG (1997). Octameric stoichiometry of the KATP channel complex. J Gen Physiol.

[155] Kline CF, Kurata HT, Hund TJ, Cunha SR, Koval OM, Wright PJ (2009). Dual role of K ATP channel C-terminal motif in membrane targeting and metabolic regulation. Proc Natl Acad Sci U S A.

[156] Levin BE, Dunn-Meynell AA, Routh VH (2001). Brain glucosensing and the K(ATP) channel. Nat Neurosci.

[157] Ainscow EK, Mirshamsi S, Tang T, Ashford ML, Rutter GA (2002). Dynamic imaging of free cytosolic ATP concentration during fuel sensing by rat hypothalamic neurones: evidence for ATP-independent control of ATP-sensitive K(+) channels. J Physiol.

[158] Lam TK, Gutierrez-Juarez R, Pocai A, Rossetti L (2005). Regulation of blood glucose by hypothalamic pyruvate metabolism. Science.

[159] Parsons MP, Hirasawa M (2010). ATP-sensitive potassium channel-mediated lactate effect on orexin neurons: implications for brain energetics during arousal. J Neurosci.

[160] Shimizu H, Watanabe E, Hiyama TY, Nagakura A, Fujikawa A, Okado H (2007). Glial Nax channels control lactate signaling to neurons for brain [Na+] sensing. Neuron.

[161] Belanger M, Allaman I, Magistretti PJ (2011). Brain energy metabolism: focus on astrocyte-neuron metabolic cooperation. Cell Metab.

[162] Liss B, Haeckel O, Wildmann J, Miki T, Seino S, Roeper J (2005). K-ATP channels promote the differential degeneration of dopaminergic midbrain neurons. Nat Neurosci.

[163] Liu D, Pitta M, Lee JH, Ray B, Lahiri DK, Furukawa K (2010). The KATP channel activator diazoxide ameliorates amyloid-beta and tau pathologies and improves memory in the 3xTgAD mouse model of Alzheimer’s disease. J Alzheimers Dis.

[164] Soundarapandian MM, Zhong X, Peng L, Wu D, Lu Y (2007). Role of K(ATP) channels in protection against neuronal excitatory insults. J Neurochem.

[165] Ashford ML, Boden PR, Treherne JM (1990). Glucose-induced excitation of hypothalamic neurones is mediated by ATP-sensitive K+ channels. Pflugers Arch.

[166] Kefaloyianni E, Bao L, Rindler MJ, Hong M, Patel T, Taskin E (2012). Measuring and evaluating the role of ATP-sensitive K+ channels in cardiac muscle. J Mol Cell Cardiol.

[167] Levin BE (2001). Glucosensing neurons do more than just sense glucose. Int J Obes Relat Metab Disord.

[168] Thomzig A, Laube G, Pruss H, Veh RW (2005). Pore-forming subunits of K-ATP channels, Kir6.1 and Kir6.2, display prominent differences in regional and cellular distribution in the rat brain. J Comp Neurol.

[169] Thomzig A, Wenzel M, Karschin C, Eaton MJ, Skatchkov SN, Karschin A (2001). Kir6.1 is the principal pore-forming subunit of astrocyte but not neuronal plasma membrane K-ATP channels. Mol Cell Neurosci.

[170] Yokoshiki H, Sunagawa M, Seki T, Sperelakis N (1998). ATP-sensitive K+ channels in pancreatic, cardiac, and vascular smooth muscle cells. Am J Physiol.

[171] Flagg TP, Enkvetchakul D, Koster JC, Nichols CG (2010). Muscle KATP channels: recent insights to energy sensing and myoprotection. Physiol Rev.

[172] Inagaki N, Gonoi T, Clement JP, Namba N, Inazawa J, Gonzalez G (1995). Reconstitution of IKATP: an inward rectifier subunit plus the sulfonylurea receptor. Science.

[173] Sakura H, Ammala C, Smith PA, Gribble FM, Ashcroft FM (1995). Cloning and functional expression of the cDNA encoding a novel ATP-sensitive potassium channel subunit expressed in pancreatic beta-cells, brain, heart and skeletal muscle. FEBS Lett.

[174] Inagaki N, Gonoi T, Clement JP, Wang CZ, Aguilar-Bryan L, Bryan J (1996). A family of sulfonylurea receptors determines the pharmacological properties of ATP-sensitive K+ channels. Neuron.

[175] Bajgar R, Seetharaman S, Kowaltowski AJ, Garlid KD, Paucek P (2001). Identification and properties of a novel intracellular (mitochondrial) ATP-sensitive potassium channel in brain. J Biol Chem.

[176] Lacza Z, Snipes JA, Kis B, Szabo C, Grover G, Busija DW (2003). Investigation of the subunit composition and the pharmacology of the mitochondrial ATP-dependent K+ channel in the brain. Brain Res.

[177] Suzuki M, Kotake K, Fujikura K, Inagaki N, Suzuki T, Gonoi T (1997). Kir6.1: a possible subunit of ATP-sensitive K+ channels in mitochondria. Biochem Biophys Res Commun.

[178] Zawar C, Plant TD, Schirra C, Konnerth A, Neumcke B (1999). Cell-type specific expression of ATP-sensitive potassium channels in the rat hippocampus. J Physiol.

[179] Hansen JB (2006). Towards selective Kir6.2/SUR1 potassium channel openers, medicinal chemistry and therapeutic perspectives. Curr Med Chem.

[180] Roseborough G, Gao D, Chen L, Trush MA, Zhou S, Williams GM (2006). The mitochondrial K-ATP channel opener, diazoxide, prevents ischemia-reperfusion injury in the rabbit spinal cord. Am J Pathol.

[181] Teshima Y, Akao M, Li RA, Chong TH, Baumgartner WA, Johnston MV (2003). Mitochondrial ATP-sensitive potassium channel activation protects cerebellar granule neurons from apoptosis induced by oxidative stress. Stroke.

[182] Wang L, Zhu QL, Wang GZ, Deng TZ, Chen R, Liu MH (2011). The protective roles of mitochondrial ATP-sensitive potassium channels during hypoxia-ischemia-reperfusion in brain. Neurosci Lett.

[183] Liu D, Lu C, Wan R, Auyeung WW, Mattson MP (2002). Activation of mitochondrial ATP-dependent potassium channels protects neurons against ischemia-induced death by a mechanism involving suppression of Bax translocation and cytochrome c release. J Cereb Blood Flow Metab.

[184] Maneuf YP, Duty S, Hille CJ, Crossman AR, Brotchie JM (1996). Modulation of GABA transmission by diazoxide and cromakalim in the globus pallidus: implications for the treatment of Parkinson’s disease. Exp Neurol.

[185] Yang YJ, Zhang S, Ding JH, Zhou F, Hu G (2009). Iptakalim protects against MPP+−induced degeneration of dopaminergic neurons in association with astrocyte activation. Int J Neuropsychopharmacol.

[186] Zhou F, Yao HH, Wu JY, Ding JH, Sun T, Hu G (2008). Opening of microglial K(ATP) channels inhibits rotenone-induced neuroinflammation. J Cell Mol Med.

[187] Goodman Y, Mattson MP (1996). K+ channel openers protect hippocampal neurons against oxidative injury and amyloid beta-peptide toxicity. Brain Res.

[188] Soundarapandian MM, Wu D, Zhong X, Petralia RS, Peng L, Tu W (2007). Expression of functional Kir6.1 channels regulates glutamate release at CA3 synapses in generation of epileptic form of seizures. J Neurochem.

[189] Virgili N, Mancera P, Wappenhans B, Sorrosal G, Biber K, Pugliese M (2013). K(ATP) channel opener diazoxide prevents neurodegeneration: a new mechanism of action via antioxidative pathway activation. PLoS One.

[190] Pugliese M, Espinosa-Parrilla JF, Virgili N, Mancera P, Pasten ZA, Mahy GJ, Rodriguez AM, Inventors 2012 Diazoxide for use in the treatment of amyotrophic lateral sclerosis. 2012. EP 2422787 A1.

[191] Alemzadeh R, Holshouser S, Massey P, Koontz J (2002). Chronic suppression of insulin by diazoxide alters the activities of key enzymes regulating hepatic gluconeogenesis in Zucker rats. Eur J Endocrinol.

[192] Kishore P, Boucai L, Zhang K, Li W, Koppaka S, Kehlenbrink S (2011). Activation of K(ATP) channels suppresses glucose production in humans. J Clin Invest.

[193] Lemak MS, Voloshanenko O, Draguhn A, Egorov AV (2014). KATP channels modulate intrinsic firing activity of immature entorhinal cortex layer III neurons. Front Cell Neurosci.

